# Normal age- and sex-based values of right ventricular free wall and four-chamber longitudinal strain by speckle-tracking echocardiography: from the Copenhagen City heart study

**DOI:** 10.1007/s00392-023-02333-x

**Published:** 2023-11-15

**Authors:** Caroline Espersen, Kristoffer Grundtvig Skaarup, Mats Christian Højbjerg Lassen, Niklas Dyrby Johansen, Raphael Hauser, Flemming Javier Olsen, Gorm Boje Jensen, Peter Schnohr, Rasmus Møgelvang, Tor Biering-Sørensen

**Affiliations:** 1 Cardiovascular Non-Invasive Imaging Research Laboratory, The Department of Cardiology, Copenhagen University Hospital - Herlev & Gentofte, Gentofte Hospitalsvej 8, 2900 Hellerup, Denmark; 2 Center for Translational Cardiology and Pragmatic Randomized Trials, Department of Biomedical Sciences, Faculty of Health and Medical Sciences, University of Copenhagen, Copenhagen, Denmark; 3 The Copenhagen City Heart Study, Copenhagen University Hospital – Bispebjerg and Frederiksberg, Copenhagen, Denmark; 4 The Department of Cardiology, Copenhagen University Hospital - Rigshospitalet, Copenhagen, Denmark

**Keywords:** Right ventricular longitudinal strain, Normal values, General population, Speckle-tracking echocardiography

## Abstract

**Aim:**

To promote the implementation of right ventricular (RV) longitudinal strain in clinical practice, we sought to propose normal values for RV free wall (RVFWLS) and four-chamber longitudinal strain (RV4CLS) and investigate the association with clinical and echocardiographic parameters in participants from the general population.

**Methods and Results:**

Participants from the 5th Copenhagen City Heart Study (2011–2015)—a prospective cohort study—with available RV longitudinal strain measurements were included. RVFWLS and RV4CLS were assessed using two-dimensional speckle-tracking echocardiography. In total, 2951 participants were included. Amongst 1297 participants without cardiovascular disease or risk factors (median age 44, 63% female), mean values of RVFWLS and RV4CLS were − 26.7% ± 5.2 (95% prediction interval (PI) − 36.9, − 16.5) and − 21.7% ± 3.4 (95%PI − 28.4, − 15.0), respectively. Women had significantly higher absolute values of RVFWLS and RV4CLS than men (mean − 27.5 ± 5.5 vs. − 25.4 ± 4.5, *p* < 0.001 and − 22.3 ± 3.5 vs. − 20.6 ± 3.0, *p* < 0.001, respectively). Absolute values of RVFWLS but not RV4CLS decreased significantly with increasing age in unadjusted linear regression. Tricuspid annular plane systolic excursion, RV s’ and left ventricular global longitudinal strain were the most influential parameters associated with both RVFWLS and RV4CLS in multiple linear regression. Participants with cardiovascular disease (*n* = 1531) had a higher proportion of abnormal values of RVFWLS and RV4CLS compared to the healthy population (8% vs. 4%, *p* < 0.001 and 8% vs. 3%, *p* < 0.001, respectively).

**Conclusion:**

This study proposed normal age- and sex-based values of RVFWLS and RV4CLS in a healthy population sample and showed significant sex differences in both measurements across ages.

**Graphical abstract:**

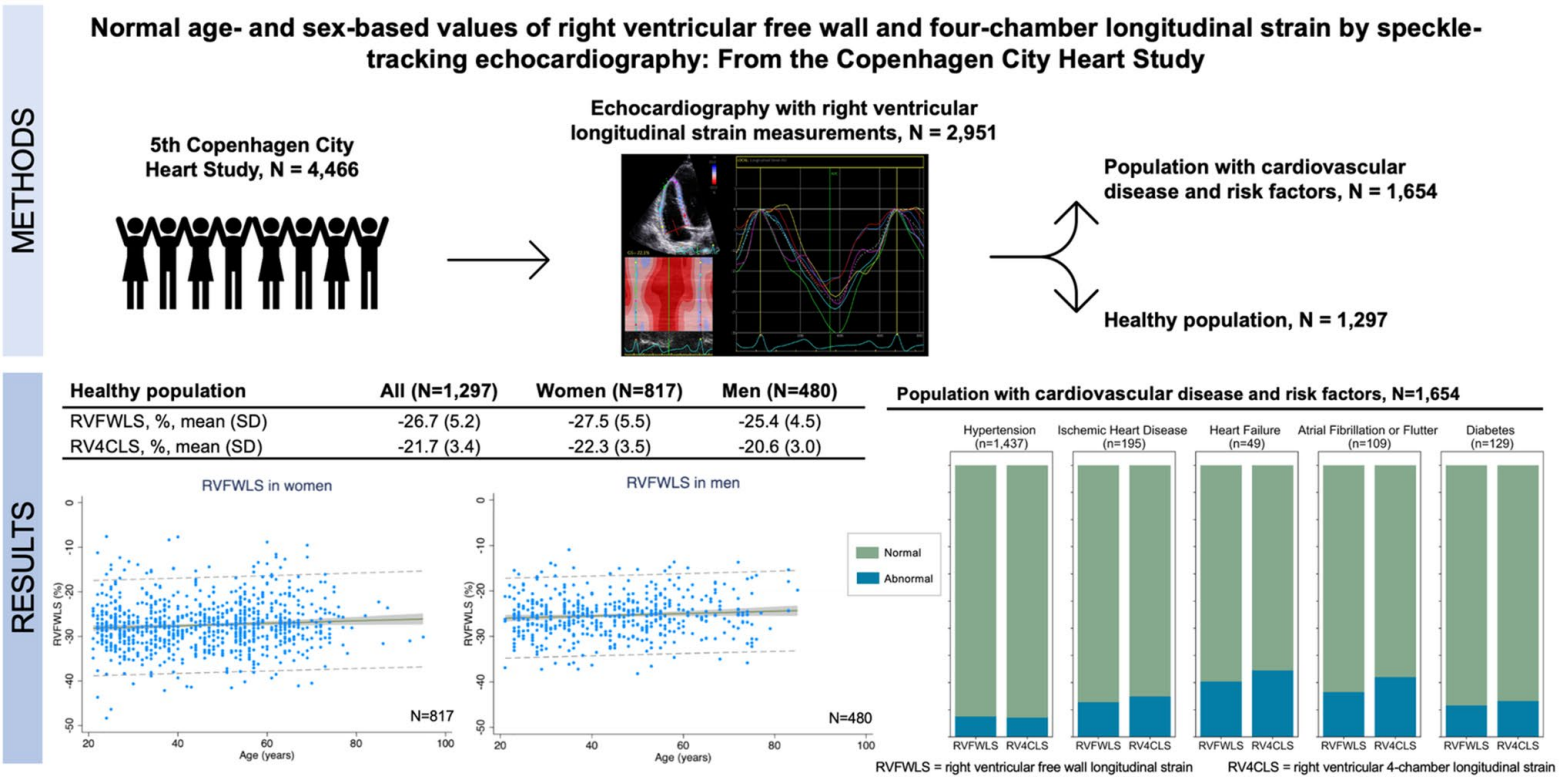

**Supplementary Information:**

The online version contains supplementary material available at 10.1007/s00392-023-02333-x.

## Introduction

Right ventricular (RV) longitudinal strain obtained with two-dimensional speckle-tracking echocardiography is a feasible and reproducible assessment of RV systolic function and may overcome some of the limitations associated with conventional RV systolic parameters by being less angle-dependent [1] and more representative of RV function [2]. RV longitudinal strain of the free wall (RVFWLS) is included as a part of the echocardiographic chamber quantification recommendations for the assessment of RV function [1]. Although RVFWLS is the recommended method for reporting RV longitudinal strain, both RVFWLS and RV four-chamber longitudinal strain (RV4CLS), with the latter including both the free wall and interventricular septum, have been proposed as novel markers of RV function [3].

Although RVFWLS and RV4CLS may offer important diagnostic and prognostic information in a range of patient groups [4–15], it has not yet been widely implemented in daily clinical echocardiographic practice when evaluating RV function. To promote wider implementation of RV longitudinal strain in clinical practice, it is important to establish normal age- and sex-based reference ranges for RVFWLS and RV4CLS. There are no definite reference ranges for RVFWLS recommended in the most recent guidelines due to lack of data from large studies [1]. Therefore, in this study, we sought to 1) propose normal age- and sex-based values of RVFWLS and RV4CLS and 2) investigate clinical and echocardiographic parameters associated with RVFWLS and RV4CLS in a large cohort of participants without cardiovascular diseases or risk factors, and 3) investigate the prevalence of abnormal RVFWLS and RV4CLS based on the proposed reference values in participants with various cardiovascular diseases and risk factors and according to conventional RV systolic parameters.

## Methods

### Study population

**Fig. 1:**
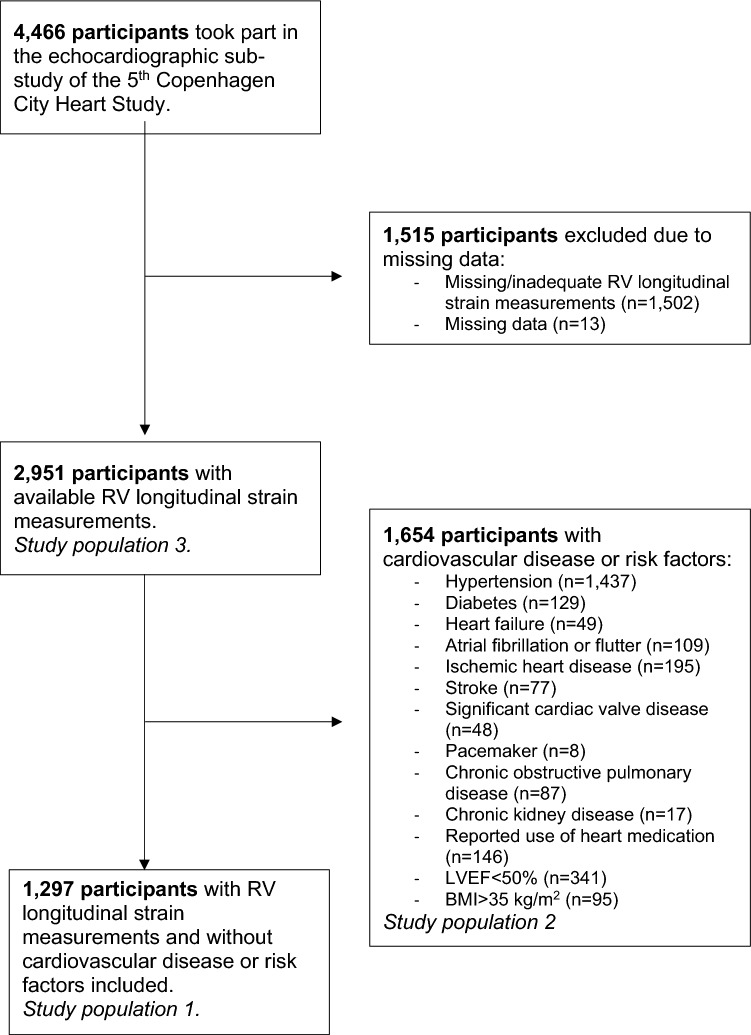
Flow chart depicting the inclusion of participants for this study

The Copenhagen City Heart Study is a prospective longitudinal cohort study assessing risk factors for cardiovascular disease in the general population (clinical trial number: NCT02993172). A total of 4466 participants took part in the 5th Copenhagen City Heart Study (2011–2015) and underwent an extensive transthoracic echocardiographic examination as well as a physical examination. Of these, 1502 participants had missing RV longitudinal strain measurements and 13 participants had missing data and were thus excluded. Therefore, in total, 2951 participants were included in the current study. To ensure a healthy study population for assessment of normal values of RV longitudinal strain, we excluded participants with the following cardiovascular and lung diseases or risk factors for cardiovascular disease at baseline (*n* = in total): hypertension (*n* = 1437), diabetes (*n* = 129), heart failure (*n* = 49), atrial fibrillation or flutter (n = 109), ischaemic heart disease (*n* = 195), stroke (*n* = 77), chronic kidney disease (*n* = 17), significant heart valve disease (*n* = 48), reported use of heart medication (*n* = 146), chronic obstructive pulmonary disease (*n* = 87), pacemaker (*n* = 8), left ventricular ejection fraction (LVEF) < 50% (*n* = 341), or a BMI > 35 kg/m^2^ (*n* = 95). Therefore, a total of 1,297 participants free of the above-mentioned cardiovascular diseases or risk factors were included in the current study to propose normal values of RVFWLS and RV4CLS (study population 1) (Fig. 1). In addition, to examine the values of RV longitudinal strain in the participants with cardiovascular risk factors or disease, we excluded the healthy participants and included those with cardiovascular or lung disease only (*n* = 1654, study population 2). Furthermore, we included the entire study population (with and without cardiovascular disease) to compare values of RV longitudinal strain to that of conventional RV systolic parameters (*n* = 2951, study population 3). Written informed consent was obtained from all participants prior to inclusion. The study was conducted in accordance with the 2nd Declaration of Helsinki and approved by the local ethics committee.

### Baseline clinical information

In addition to the physical examination, all participants filled out an extensive self-administered questionnaire regarding their overall health status including prescribed medication, physical activity and smoking status. They furthermore underwent non-fasting blood tests. Information on comorbidites including heart failure, ischaemic heart disease, stroke, pacemaker implantation, chronic obstructive pulmonary disease, and chronic kidney disease was obtained from the Danish National Patient Registry using ICD-8/10 codes. Atrial fibrillation or flutter was defined as an ICD-8/10 diagnostic code for atrial fibrillation or flutter, or atrial fibrillation or flutter during the echocardiographic examination. The definitions of hypertension and diabetes have been described in detail previously [16]. Information on valve disease (mitral or aortic valve disease) was obtained from the Danish National Patient Registry using ICD-8/10 codes and the presence of aortic stenosis, severe aortic regurgitation or moderate to severe mitral regurgitation on the echocardiogram. A detailed definition of relevant comorbidities can be found in the Supplemental Material.

### Echocardiography

All echocardiographic examinations were performed by experienced sonographers using GE Vivid 9 ultrasound machines (GE Healthcare, Horten, Norway) according to a standardised protocol. The echocardiograms were analysed offline in accordance with recommended guidelines [1] with commercially available software (EchoPAC v. 113, GE Healthcare) by an experienced investigator blinded to clinical data.

#### Conventional echocardiography

The measurements obtained from the conventional echocardiographic examination included left ventricular (LV) dimensions, left ventricular ejection fraction (LVEF), left atrial volume, transmitral inflow velocity and mitral annular velocity. The detailed acquisition of these parameters has been described previously [16, 17]. In addition to these measurements, RV functional parameters were analysed, including tricuspid annular plane systolic excursion (TAPSE), fractional area change (FAC), tricuspid lateral annular systolic velocity (RV S’) and the estimated right ventricular systolic pressure (RVSP). All RV measurements were performed in the RV focused apical 4-chamber view. A detailed description of how the conventional RV conventional parameters were obtained can be found in the Supplemental Material.

#### Two-dimensional speckle-tracking echocardiography

**Fig. 2:**
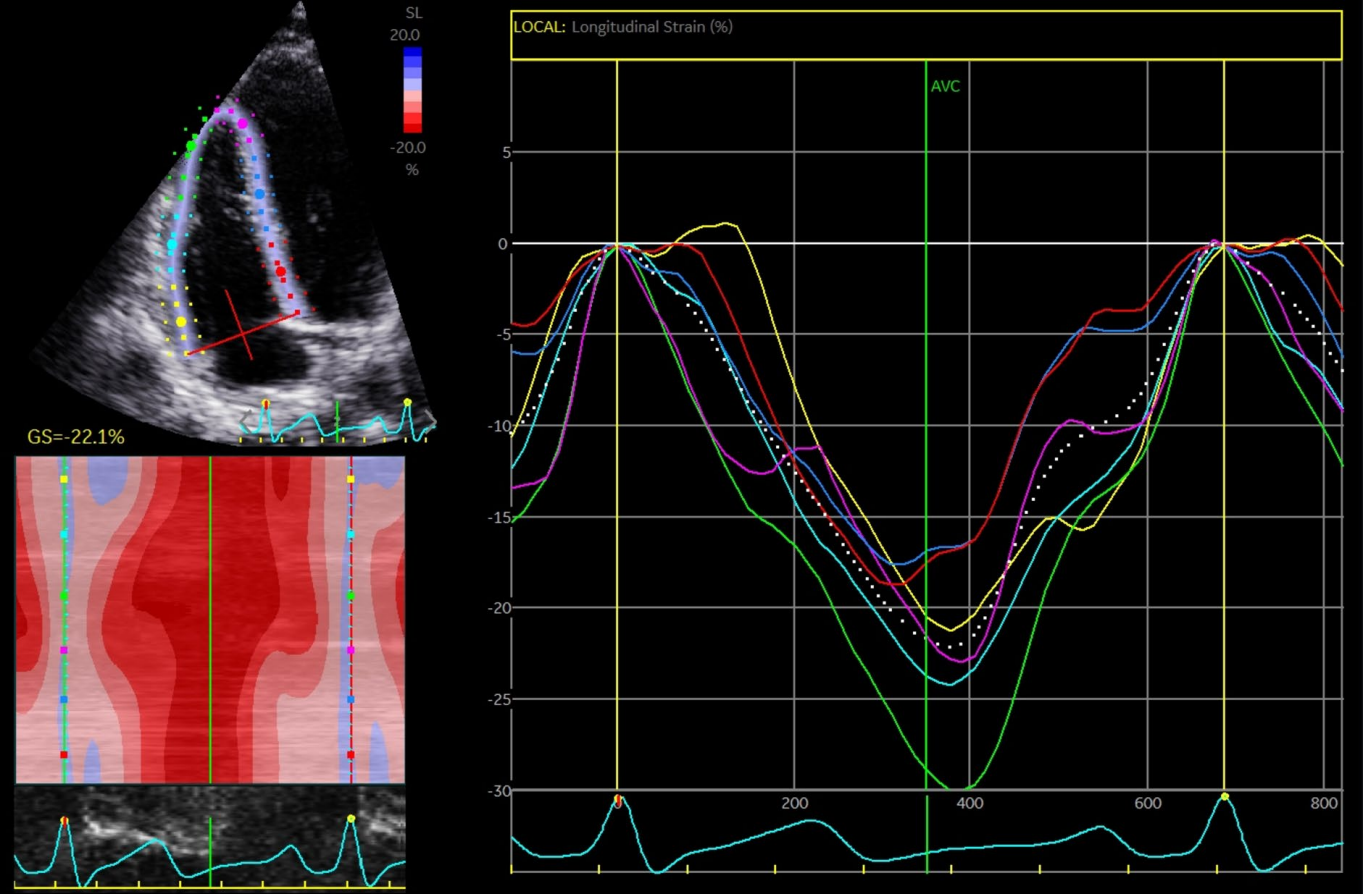
Example of two-dimensional speckle-tracking of the RV for obtaining RV4CLS

Two-dimensional speckle-tracking of the RV was performed in a semi-automated fashion in a RV focused apical 4-chamber view. The software automatically traced the endocardial border and generated a region of interest covering the entire myocardial wall. The region of interest could be adjusted manually if the tracing was deemed inadequate. In case of persistently inaccurate tracing, segments could be excluded. The longitudinal strain values were calculated as the average value of the peak longitudinal strain values from all three or six segments in the region of interest for RVFWLS and RV4CLS, respectively. RV4CLS was calculated from the segments in both the RV free and septal wall (Fig. 2), whereas the RVFWLS was calculated from the segments in the RV free wall only. The frame rate was above 40 frames/s for the two-dimensional speckle-tracking analysis.

To ensure adequate RV strain measurements, RV longitudinal strain data were checked for outliers and if RVFWLS or RV4CLS > -5%, the echocardiographic images were reviewed again by a second investigator. If these analyses were considered incorrect, the observations were dropped.

LV global longitudinal strain (GLS) was obtained from six regional segments in all three apical views as has been described in detail previously [16].

### Statistical analysis

Continuous variables were listed with mean and standard deviation or with median and interquartile range (IQR) for normally and non-normally distributed variables, respectively. Normally distributed variables were compared using the Student’s t test, whereas non-normally distributed variables were compared using the Wilcoxon rank-sum test. Categorical variables were listed with absolute numbers and percentages and compared using chi-square test.

The reference ranges for RV longitudinal strain parameters are presented for the overall study sample and stratified by sex and age categories. As both RVFWLS and RV4CLS were normally distributed, they were listed with mean and standard deviation as well 95% prediction intervals (PI) defined by mean ± 1.96 * standard deviation (SD). We assessed trends in RV longitudinal strain parameters across age categories using linear regression. Absolute values of RVFWLS and RV4CLS below (mean - 1.96*SD) were considered abnormal. Initially, cubic spline models were used to assess whether the association between age and RV longitudinal strain was linear or non-linear. As there was no evidence of a non-linear association, unadjusted linear regression analyses were performed to investigate the association between age, and RVFWLS and RV4CLS. Tests for interaction were performed to assess for effect modification of sex and age on RVFWLS and RV4CLS. To examine clinical and echocardiographic parameters correlated with RVFWLS and RV4CLS, we performed unadjusted and multivariable linear regression analyses. The multivariable linear regression analyses were adjusted for age, sex, smoking status, physical activity, heart rate, systolic blood pressure, RVSP, and LVEF, and the standardised beta-coefficients were reported. In the population with cardiovascular disease and risk factors, we investigated the proportion of participants with abnormal values of RVFWLS and RV4CLS. Likewise, we assessed the proportion of participants with abnormal values of RVFWLS and RV4CLS in participants with normal and abnormal values of conventional RV systolic parameters (TAPSE, RV FAC, and RV S’) in the whole cohort with or without cardiovascular disease or risk factors. Abnormal cut-off values for TAPSE (< 17 mm), RV FAC (< 35%) and RV S’ (< 0.095) were based on the current recommendations [1]. The intra- and inter-observer variability of RVFWLS and RV4CLS was assessed in 10 randomly selected subjects (Supplemental Material). Mean difference ± SD and individual intra-class correlation coefficients (ICC) were reported. A two-sided *p* value of < 0.05 was considered statistically significant. All statistical analyses were performed in STATA/SE version 17.

## Results

### Baseline characteristics of all included study participants and stratified by sex

The baseline characteristics of all included study participants and stratified according to sex are listed in Table 1. Median age of the participants was 44 years (IQR 32–56) and 817 (63%) were female. The median blood pressure was 124/74 mmHg, and the mean BMI was 24 kg/m^2^. The median frame rate for the speckle-tracking images was 63 frames/second. Mean LVEF and TAPSE were 59% and 27.0 mm, respectively. Overall, women had lower blood pressure, lower BMI, and were less physically active in their leisure time than men, whereas men had lower heart rate, higher creatinine, LDL cholesterol, and haemoglobin. Women presented with better LV systolic function including higher LVEF and absolute LV GLS, and women also had higher values of E/e’. Men had higher TAPSE, RV s’, TR velocity, and RVSP but lower RV FAC compared to women.

**Table 1:** Baseline characteristics of the population stratified according to sex

	N	Total	Women	Men	*P*
	1297	N = 1297	*N* = 817	*N* = 480	
Demographics and clinical characteristics					
Age, years	1297	44 (32–56)	46 (32–57)	43 (32–55)	0.16
Systolic blood pressure, mmHg	1297	124 (116–131)	121 (113–128)	128 (121–133)	< 0.001
Diastolic blood pressure, mmHg	1297	74 (69–79)	74 (68–79)	75 (70–79)	0.002
Heart rate, bpm	1277	63 (10)	63 (10)	61 (10)	< 0.001
BMI, kg/m^2^	1297	24 (3)	23 (3)	24 (3)	< 0.001
BSA, m^2^	1297	1.83 (0.19)	1.74 (0.15)	1.99 (0.15)	< 0.001
Smoking status	1239				0.12
Never		577 (46.6%)	361 (46.4%)	216 (46.9%)	
Previous		446 (36.0%)	293 (37.7%)	153 (33.2%)	
Active		216 (17.4%)	124 (15.9%)	92 (20.0%)	
Physical activity in leisure time	1289				< 0.001
Sedentary, *n* (%)		53 (4.1%)	36 (4.4%)	17 (3.6%)	
Low, *n* (%)		396 (30.7%)	267 (32.8%)	129 (27.2%)	
Moderate, *n* (%)		681 (52.8%)	449 (55.2%)	232 (48.8%)	
High, *n* (%)		159 (12.3%)	62 (7.6%)	97 (20.4%)	
Laboratory work					
Total cholesterol, mmol/L	1265	5.2 (1.1)	5.2 (1.1)	5.1 (1.0)	0.090
LDL cholesterol, mmol/L	1265	2.9 (0.9)	2.9 (0.9)	3.0 (0.9)	0.023
HDL cholesterol, mmol/L	1265	1.6 (0.5)	1.8 (0.5)	1.4 (0.4)	< 0.001
Glucose, mmol/L	1260	5.1 (0.8)	5.1 (0.7)	5.2 (1.0)	< 0.001
Creatinine, umol/L	1256	74.4 (11.1)	70.0 (9.1)	81.5 (10.4)	< 0.001
Haemoglobin, mmol/L	1281	8.7 (0.7)	8.4 (0.6)	9.2 (0.6)	< 0.001
Echocardiography					
LVEDV, mL	1277	109.3 (27.6)	96.7 (18.8)	130.8 (26.9)	< 0.001
LVESV, mL	1277	45.5 (12.7)	39.5 (8.5)	55.6 (12.3)	< 0.001
LVSV, mL	1277	63.9 (16.4)	57.2 (12.0)	75.2 (16.7)	< 0.001
LVEF, %	1235	59 (4)	59 (4)	58 (4)	< 0.001
GLS, %	1278	-20.6 (1.9)	-21.1 (1.8)	-19.6 (1.8)	< 0.001
LVMI, g/m^2^	1283	79.6 (16.5)	74.0 (13.9)	89.1 (16.2)	< 0.001
LAVI, mL/m^2^	1293	23.6 (6.9)	23.1 (6.7)	24.4 (7.2)	0.001
E/A	1267	1.4 (1.1–1.9)	1.5 (1.1–1.9)	1.4 (1.1–1.8)	0.035
E/e’ (average)	1259	5.9 (5.1–7.1)	6.1 (5.2–7.4)	5.6 (4.8–6.6)	< 0.001
DT, ms	1266	187.6 (38.3)	185.1 (37.6)	191.9 (39.2)	0.002
TAPSE, mm	1260	27.0 (4.0)	26.5 (3.9)	27.9 (4.1)	< 0.001
RV S’, m/s	1246	0.14 (0.02)	0.14 (0.02)	0.15 (0.02)	< 0.001
FAC, %	1189	35.1 (8.3)	35.9 (8.2)	33.8 (8.4)	< 0.001
TR velocity, m/s	757	− 2.19 (0.23)	− 2.17 (0.22)	− 2.22 (0.24)	0.011
RVSP, mmHG	757	25.0 (5.4)	24.6 (5.3)	25.7 (5.6)	0.016

*BMI* body mass index, *BSA* body surface area, *LDL* low density lipoprotein, *HDL* high density lipoprotein, *LVESV* left ventricular end-systolic volume, *LVEDV* left ventricular end-diastolic volume, *LVSV* left ventricular stroke volume, *LVEF* left ventricular ejection fraction, *GLS* global longitudinal strain, *LVMI* left ventricular mass index, *LAVI* left atrial volume index, *DT* deceleration time, *TR* tricuspid regurgitation, *TAPSE* tricuspid annular plane systolic excursion, *FAC* fractional area change, *RVSP* right ventricular systolic pressure

### Normal values of RVFWLS and RV4CLS according to sex and age

The normal reference values including 95% PI of RVFWLS and RV4CLS are listed for the whole study population and stratified by sex in Table 2. The mean values of RVFWLS and RV4CLS were − 26.7% ± 5.2 (95% PI − 36.9, − 16.5) and − 21.7% ± 3.4 (95%PI − 28.4, − 15.0) in the entire population. The absolute values of RVFWLS and RV4CLS were significantly higher in women as compared to men (mean − 27.5 ± 5.5 vs. − 25.4 ± 4.5, *p* < 0.001 and − 22.3 ± 3.5 vs. − 20.6 ± 3.0, *p* < 0.001, respectively).

**Table 2:** RV longitudinal strain parameters according to age and sex

Age range, years	All	21–39	40–59	≥ 60	*P* for trend
Entire healthy study population	***N* = 1297**	***N* = 548**	***N* = 500**	***N* = 288**	
RVFWLS, % SD (PI)	− 26.7 ± 5.2 (− 36.9, − 16.5)	− 27.0 ± 5.3 (− 37.1, − 16.6)	− 26.8 ± 4.9 (− 36.2, − 17.3)	− 25.9 ± 5.6 (− 36.6, − 14.3)	0.008
RV4CLS, % SD (PI)	− 21.7 ± 3.4 (− 28.4, − 15.0)	− 21.8 ± 3.4 (− 28.5, − 14.8)	− 21.7 ± 3.3 (− 27.9, − 15.3)	− 21.3 ± 3.6 (− 28.4, − 14.2)	0.07
Women	*N* = 817	*N* = 336	*N* = 310	*N* = 171	
RVFWLS, % SD (PI)	− 27.5 ± 5.5* (− 38.1, − 16.7)	− 27.9 ± 5.5* (− 38.7, − 17.0)	− 27.6 ± 5.1* (− 37.5, − 17.6)	− 26.5 ± 5.9* (− 38.0, − 14.9)	0.008
RV4CLS, % SD (PI)	− 22.3 ± 3.5* (− 29.2, − 15.4)	− 22.5 ± 3.4* (− 29.3, − 15.8)	− 22.4 ± 3.4* (− 29.0, -15.8)	− 21.8 ± 3.8* (− 29.3, − 14.3)	0.049
Men	*N* = 480	*N* = 212	N = 190	*N* = 78	
RVFWLS, % SD (PI)	− 25.4 ± 4.5* (− 34.2, − 16.6)	− 25.6 ± 4.5* (− 34.4, − 16.8)	− 25.5 ± 4.3* (− 34.0, − 17.0)	− 24.6 ± 4.7* (− 33.8, − 15.4)	0.15
RV4CLS, % SD (PI)	− 20.6 ± 3.0* (− 26.4, − 14.8)	− 20.7 ± 3.0* (− 26.6, − 14.9)	− 20.6 ± 2.9* (− 26.3, − 14.9)	− 20.3 ± 2.9* (− 26.0, − 14.5)	0.25

^*^Statistically significant difference (*p* < 0.05) for comparison between women and men for both RVFWLS and RV4CLS in the total population and across each age group

*RVFWLS* right ventricular free wall longitudinal strain, *RV4CLS* right ventricular four-chamber longitudinal strain

**Fig. 3:**
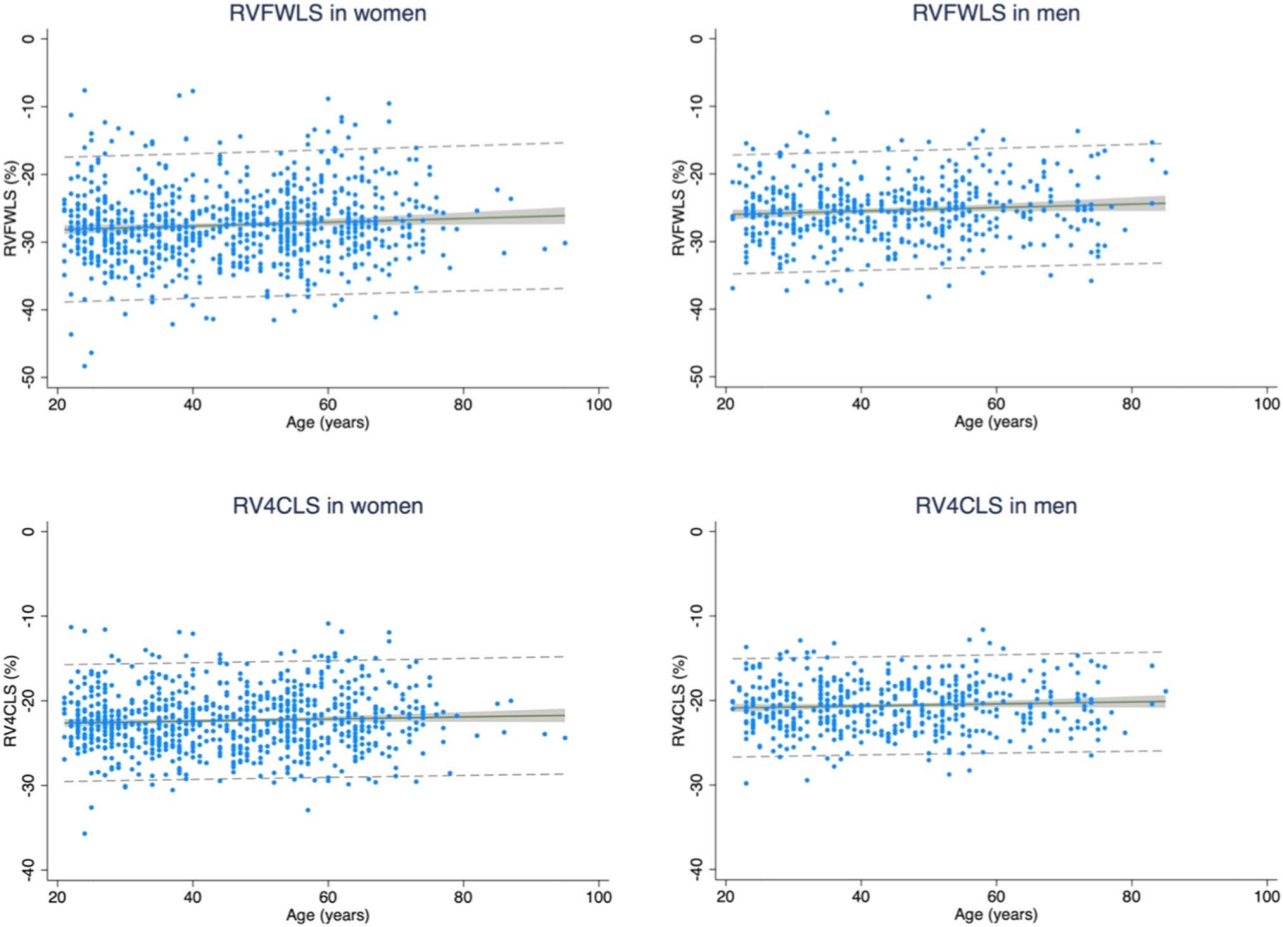
Reference values of RV free wall and four-chamber longitudinal strain across ages in women and men**.** The regression line is depicted in black, and the 95% confidence interval of the mean is depicted in grey. The 95% confidence interval for the individual forecasts including both the uncertainty of the mean prediction and the residual is illustrated with the dashed grey line. *RV: right ventricular*

We observed a significant trend in decreasing values of RVFWLS with increasing age category (*p* for trend 0.008). In unadjusted linear regression, RVFWLS decreased with an absolute value of 0.25% per 10 years increase in age, *p* = 0.009. Although the absolute values of RV4CLS did decrease across age categories, this decrease was not statistically significant (*p* for trend 0.07). Sex did not modify the association between age and RVFWLS or RV4CLS (*p* for interaction: 0.92 and 0.98, respectively). Table 2 lists the sex-stratified values of RVFWLS and RV4CLS and the corresponding 95% PI across age categories. We observed a significant trend in decreasing absolute values of RVFWLS and RV4CLS across the age categories in women but not men. Absolute values of RVFWLS and RV4CLS were significantly higher in women than men across all age categories. Figure 3 depicts values of RVFWLS and RV4CLS according to age in women and men separately.

**Table 3:** Clinical and echocardiographic parameters associated with RV free wall longitudinal strain in the healthy study population (*N* = 1297)

	Univariable		Multivariable*	
	Standardised ß-coefficient	*P*	Standardised ß-coefficient	*P*
Clinical parameters				
Age, years	− 0.07	0.009	− 0.04	0.28
Systolic blood pressure, mmHg	− 0.12	< 0.001	− 0.02	0.65
BMI, kg/m^2^	− 0.21	< 0.001	− 0.14	< 0.001
Total cholesterol, mmol/L	− 0.09	0.001	− 0.04	0.34
Heart rate, bpm	− 0.05	0.06	0.02	0.68
Echocardiographic parameters				
LVEF, %	0.17	< 0.001	0.13	0.001
GLS, %	0.29	< 0.001	0.21	< 0.001
LVMI, g/m^2^	− 0.05	0.05	0.06	0.13
LAVI, mL/m^2^	0.06	0.046	0.07	0.06
E/A	0.13	< 0.001	0.08	0.16
E/e’	− 0.02	0.46	− 0.07	0.16
TR velocity, m/s	− 0.07	0.06	− 0.02	0.76
TAPSE, mm	0.22	< 0.001	0.27	< 0.001
RV S’, m/s	0.23	< 0.001	0.25	< 0.001
FAC, %	0.09	0.003	0.08	0.049
RVSP, mmHg	0.10	0.007	0.12	0.001

*BMI* body mass index, *LVEF* left ventricular ejection fraction, *GLS* global longitudinal strain, *LVMI* left ventricular mass index, *LAVI* left atrial volume index, *TR* tricuspid regurgitation, *TAPSE* tricuspid annular plane systolic excursion, *FAC* fractional area change, *RVSP* right ventricular systolic pressure

*Adjusted for age, gender, smoking status, physical activity, heart rate, systolic blood pressure, RVSP and LVEF

**Strain values were considered as absolute values

### Clinical and echocardiographic parameters associated with RV longitudinal strain

In unadjusted linear regression analyses, several baseline clinical parameters, including age, BMI, systolic blood pressure, total cholesterol, as well as several echocardiographic parameters were significantly associated with RVFWLS and RV4CLS (Table 3 and Table S1 in the Supplemental Material). When adjusting for baseline clinical variables, RVSP and LVEF; TAPSE, RV S’, and LV GLS were the most influential parameters of RVFWLS and RV4CLS (absolute value of standardised beta-coefficients ≥ 0.20). In addition, LVEF, FAC and RVSP were associated with both RV4CLS and RVFWLS in adjusted analyses. Amongst the clinical parameters, only increasing BMI remained associated with decreasing absolute RVFLWS and RV4CLS in adjusted analyses.

### RV longitudinal strain according to cardiovascular disease, risk factors and RV conventional systolic parameters

**Fig. 4:**
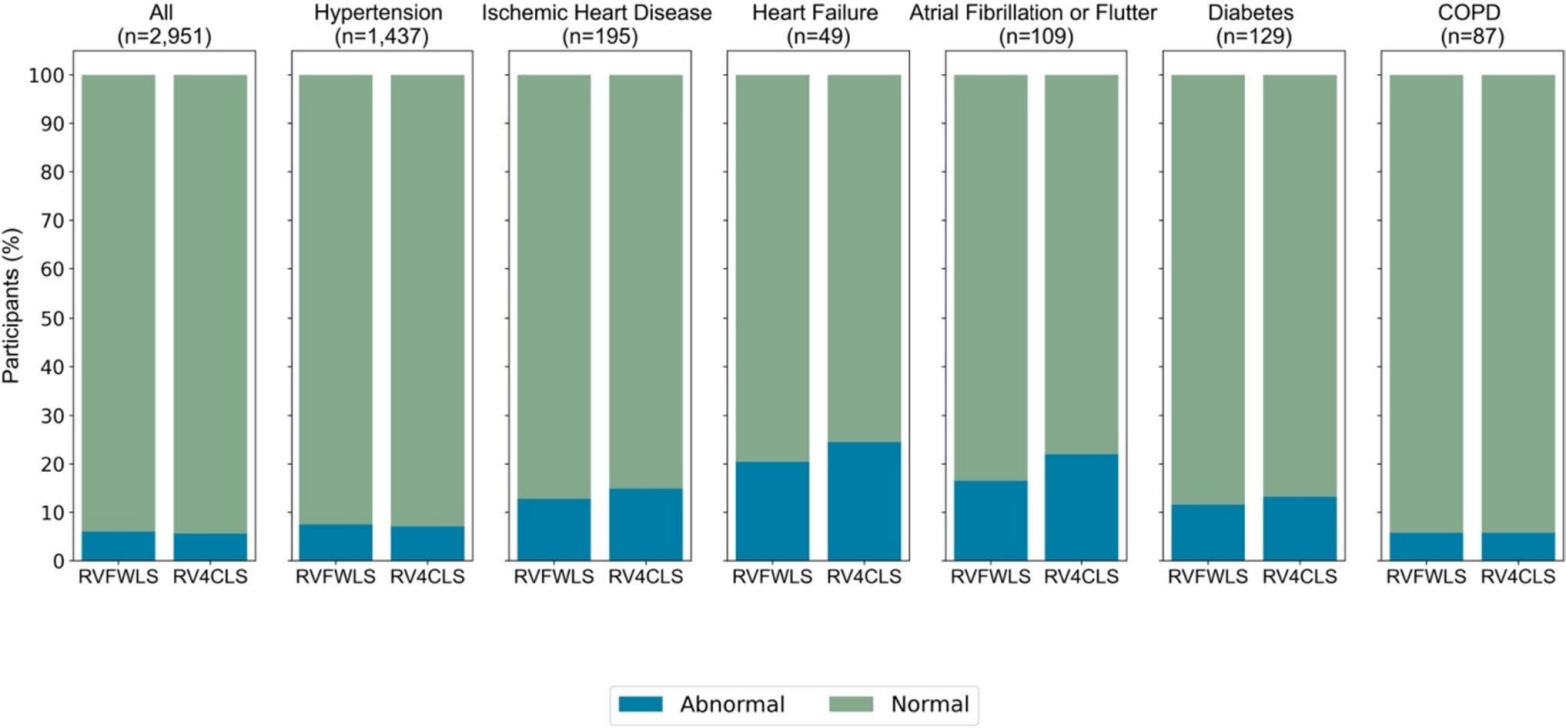
RV free wall and four-chamber longitudinal strain in participants with cardiovascular disease and risk factors. The figure depicts the proportion of participants with abnormal and normal RV free wall and four-chamber longitudinal strain in all participants and those with hypertension, ischaemic heart disease, heart failure, atrial fibrillation or flutter, diabetes and chronic obstructive pulmonary disease. The blue colour corresponds to the participants with abnormal values, whereas the green colour corresponds to the participants with normal values. *RV* right ventricular, *COPD *chronic obstructive pulmonary disease

**Fig. 5:**
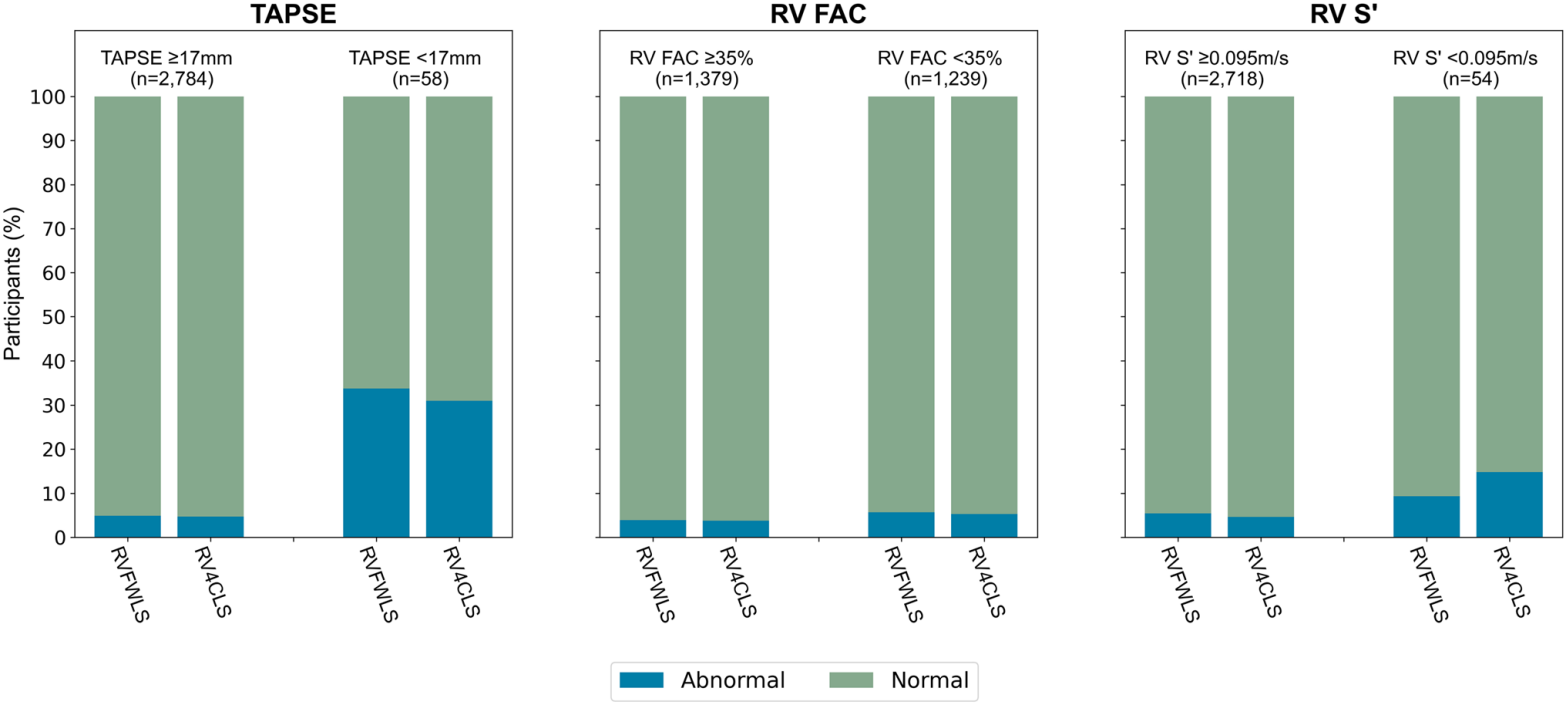
RV free wall and four-chamber longitudinal strain according to conventional RV systolic parameters. The figure depicts the proportion of participants with abnormal and normal RV free wall and four-chamber longitudinal strain in participants with normal and abnormal TAPSE, RV FAC and RV S’. The blue colour corresponds to the participants with abnormal RVFWLS or RV4CLS values, whereas the green colour corresponds to the participants with normal values of RVFWLS and RV4CLS. *RV* right ventricular, *FAC* fractional area change

Considering the population with cardiovascular disease and risk factors (Study population 2, *n* = 1654), there was a significantly higher proportion of participants with abnormal RVFWLS and RV4CLS compared to the healthy population sample (*n* = 1297) (7.9% vs. 3.6%, *p* < 0.001 and 7.7% vs. 3.0%, *p* < 0.001, respectively). Moreover, when stratified according to cardiovascular disease or risk factor (Fig. 4), participants with heart failure (*n* = 49) had the highest proportion of participants with abnormal RVFWLS and RV4CLS (20.4% and 24.5%, respectively). In the entire population with available RV longitudinal strain data (Study population 3, *n* = 2951), we found that in participants with normal TAPSE (≥ 17 mm), normal FAC (≥ 35%) and normal RV S’ (≥ 0.10 m/s), a small proportion had abnormal RVFWLS and RV4CLS (4–5%) (Fig. 4). Amongst participants with abnormal TAPSE (< 17 mm), FAC (< 35%) and RV S’ (< 0.10 m/s), there was a high proportion of participants with normal RVFWLS and RV4CLS (Fig. 5).

## Discussion

This is one of the largest prospective studies proposing normal age- and sex-based reference values of RVFWLS and RV4CLS in a general population sample without cardiovascular disease or risk factors. Overall, we determined normal values and lower and upper limits for RVFWLS and RV4CLS in the whole cohort and across sex and age categories. RVFWLS and RV4CLS were significantly higher in women as compared to men and this difference was observed throughout the age categories. Moreover, absolute values of RVFWLS decreased significantly with increasing age, but we did not find a significant decrease in RV4CLS with increasing age. There was no significant interaction between age and sex on either RV longitudinal strain parameter. Amongst the echocardiographic parameters, TAPSE, RV S' and GLS were the most influential parameters associated with both RVFWLS and RV4CLS.

Prior studies (*n* = 116 to 1913) have also investigated and proposed normal reference ranges of RVFWLS and RV4CHLS in healthy subjects [8, 18–21]. All these studies used GE Healthcare software for the echocardiographic analyses [8, 18–20], except for the WASE study which used multivendor software (including Philips, Siemens and GE) [21]. Overall, the study populations were similar in terms of mean age (range 36.5 to 48) and sex distribution (49–58% women). However, the studies differ in the population exclusion criteria. Overall, our reported mean values of RVFWLS and RV4CLS are in accordance with the findings of the NORMAL study and the Mayo Clinic Echocardiography Laboratory Study (RVFWLS only). The NORMAL study (*n* = 493, mean age 47, 53% women) reported mean RVFWLS and RV4CLS of − 26.4% ± 4.2 and − 21.5% ± 3.2, respectively, corresponding to a reference range (± 1.96 SDs) of -18.2% to -34.6% for RVFWLS and 15.2% to − 27.8% for RV4CLS [19]. The Mayo Clinic Echocardiography Laboratory (*n* = 116, mean age 48, 58% women) reported a mean RVFWLS of − 26% ± 4 with a corresponding reference range (± 2 SDs) of − 18% to − 34%[20]. The WASE study (*n* = 1913, mean age 47, 49% women) reported the highest mean values and lower limits of normality (2.5th percentile) of both RVFWLS and RV4CLS of − 28.3% ± 4.3 (− 20.0%) and − 25.4% ± 3.8 (− 18.2%), respectively [21]. In addition, a recent meta-analysis including 45 studies with a total of 4439 subjects (mean age range 23–67 years, 33–66% men) reported a pooled mean and lower limit of normality (− 1.96 SDs) for RVFWLS and RV4CLS of − 26.9% (− 18.0%) and − 23.4% (− 16.4%) [22]. Although our reported mean value of RVFWLS and RV4CLS are similar to previous studies, we reported the lowest limits of normality of both RVFWLS and RV4CLS. There are several aspects that may explain these differences including different age spectrum, gender composition and definition of the healthy study population. Our study included a high number of participants (*n* = 1297) from a broad age spectrum (ages 21–95) with a predominantly female composition from a homogenous community-based cohort. Approximately 22% of participants were ≥ 60 years of age, whereas the age range of the included participants of the NORMAL study and Mayo Clinical Echocardiography Laboratory study was 19 to 79 overall [19, 20]. Moreover, the exclusion criteria of some of the other studies were stricter, e.g. excluding participants with dyslipidemia, estimated glomerular filtration < 60 ml/min/1.73m^2^, professional sport activities, etc. In our study, we excluded participants based on important baseline cardiovascular and pulmonary diseases and risk factors as well as LVEF < 50% and presence of significant valve disease on the echocardiogram.

### RV strain values according to age and sex and influential parameters of RV function

In accordance with prior studies [18, 19, 21, 23], we found that absolute values of RVFWLS and RV4CLS were significantly higher in women compared to men. However, unlike the NORMAL echocardiography study, in which the sex difference was not evident in the older group (> 50 years) [19], the sex difference was observed throughout the age categories in our study. Although the results regarding age-related changes in RV strain are heterogeneous [8, 19], only RVFWLS decreased significantly with increasing age in our study and there was no significant interaction between sex and age on RVFWLS and RV4CLS. Moreover, when adjusting for clinical and echocardiographic parameters, age was no longer significantly associated with RVFWLS and RV4CLS in multivariable linear regression, suggesting that the age-related changes in RV longitudinal strain are subtle.

Higher values of conventional measures of RV systolic function including TAPSE, RV S’ and FAC, were associated with higher absolute values of RVFWLS and RV4CLS when adjusting for baseline clinical variables, RVSP and LVEF. A recent meta-analysis also found that higher RV FAC was associated with higher absolute RV strain parameters. However, they did not find a significant association between RV S’ and TAPSE and either RV strain parameter [22]. In addition, parameters of LV function, including GLS and LVEF, were also associated with both RVFWLS and RV4CLS in adjusted analyses in our study. This may in part be explained by interventricular dependency and the strong association between LV and RV systolic function. Moreover, GLS measurements also include the interventricular septum and is thus invariably linked to RV4CLS and to some extent also RVFWLS. Interestingly, increasing BMI was associated with lower RVFWLS and RV4CLS, possibly explained by multifactorial mechanisms and risk factors associated with higher BMI.

### RV longitudinal strain according to cardiovascular disease and risk factors

As expected, we found that there was a significantly higher proportion of participants with abnormal values of RVFWLS and RV4CLS in the participants with cardiovascular disease and risk factors compared to those without (8% vs. 4 or 3%, respectively). Moreover, when stratifying according to individual risk factors, we found that participants with heart failure had the highest proportion of participants with abnormal RVFWLS and RV4CLS (20% and 25%, respectively). These numbers are similar to that of the study by Carluccio et al. in 200 patients with heart failure with reduced ejection fraction in which they found that 25% had impaired RVFWLS (≥ − 15.3%)[24]. This underscores the potential of RV longitudinal strain for risk prediction in patients with or at risk of heart failure. Interestingly, we found that a large proportion of participants with abnormal conventional RV systolic parameters, including TAPSE, RV FAC and RV S’, had RVFWLS and RV4CLS values within the normal range based on this cohort, thus suggesting a discrepancy in the conventional systolic markers and RV longitudinal strain. This may further highlight the value of incorporating RV longitudinal strain into the standard assessment of RV function to improve identification and classification of patients with reduced RV systolic function. However, in addition to longitudinal shortening, radial and anteroposterior systolic motion of the RV contribute to RV pump function. As RV longitudinal strain measurements only incorporate longitudinal motion of the RV, these measurements do not consider the effect of radial and anteroposterior motion on RV function [25].

Although RVFWLS remains the recommended RV strain assessment [1, 3], there is not yet universal consensus regarding the use of RVFWLS or RV4CLS for the assessment of RV function with speckle-tracking imaging. It is argued that RV4CLS may be influenced by LV dysfunction [6] as it includes the interventricular septum and that it is therefore a less accurate marker of RV systolic function. In our study, we have reported age- and sex-stratified values of both RVFWLS and RV4CLS and clinical and echocardiographic parameters associated with both. Additional studies are needed in order to determine which strain parameter best reflects RV function.

## Limitations

The results of our study should be interpreted considering the limitations. First, we only used one type of software and ultrasound vendor for the echocardiography and subsequent analysis. This may affect the generalizability of our results due to substantial inter-vendor variability in RV longitudinal strain measurements [3]. There was also a substantial number of missing RV strain measurements due to poor image quality, but even so, this is still one of the largest studies reporting normal reference values of RV longitudinal strain in participants from the general population free of cardiovascular disease and risk factors. Moreover, this was a predominantly homogeneous, Caucasian population, which may also limit the generalizability of the reference values to other populations and ethnicities.

## Conclusion

In this large prospective study of healthy participants from the general population, we have proposed age- and sex-based normal reference material for RV longitudinal strain parameters, including RVFWLS and RV4CLS. Overall, women had higher absolute values of RVFWLS and RV4CLS than men, and absolute values of RVFWLS decreased significantly with increasing age. TAPSE, RV S’ and LV GLS were the most influential parameters associated with both RVFWLS and RV4CLS.

### Supplementary Information

Below is the link to the electronic supplementary material.

Supplementary file1 (DOCX 27 kb)

## Data Availability

The data underlying this article cannot be shared publicly due to the privacy of the individuals who participated in the study. The data will be shared on reasonable request to the corresponding author.

## References

[1] Lang RM, Badano LP, Mor-Avi V, Afilalo J, Armstrong A, Ernande L (2015). Recommendations for cardiac chamber quantification by echocardiography in adults: an update from the American society of echocardiography and the European association of cardiovascular imaging. Europ Heart J Cardiovascular Imag.

[2] Giusca S, Dambrauskaite V, Scheurwegs C, D'hooge J, Claus P, Herbots L, (2010). Deformation imaging describes right ventricular function better than longitudinal displacement of the tricuspid ring. Heart.

[3] Badano LP, Muraru D, Parati G, Haugaa K, Voigt J-U (2020). How to do right ventricular strain. Europ Heart J Cardiovas Imag.

[4] Cameli M, Righini FM, Lisi M, Bennati E, Navarri R, Lunghetti S (2013). Comparison of right versus left ventricular strain analysis as a predictor of outcome in patients with systolic heart failure referred for heart transplantation. Am J Cardiol.

[5] Hamada-Harimura Y, Seo Y, Ishizu T, Nishi I, Machino-Ohtsuka T, Yamamoto M (2018). Incremental prognostic value of right ventricular strain in patients with acute decompensated heart failure. Circulation Cardiovas Imag.

[6] Carluccio E, Biagioli P, Lauciello R, Zuchi C, Mengoni A, Bardelli G (2019). Superior prognostic value of right ventricular free wall compared to global longitudinal strain in patients with heart failure. J Am Soc Echocardiogr.

[7] Bosch L, Lam CSP, Gong L, Chan SP, Sim D, Yeo D (2017). Right ventricular dysfunction in left-sided heart failure with preserved versus reduced ejection fraction. Eur J Heart Fail.

[8] Morris DA, Krisper M, Nakatani S, Köhncke C, Otsuji Y, Belyavskiy E (2016). Normal range and usefulness of right ventricular systolic strain to detect subtle right ventricular systolic abnormalities in patients with heart failure: a multicentre study. Europ Heart J Cardiovas Imag.

[9] Gavazzoni M, Badano LP, Vizzardi E, Raddino R, Genovese D, Taramasso M (2019). Prognostic value of right ventricular free wall longitudinal strain in a large cohort of outpatients with left-side heart disease. Europ Heart J Cardiovas Imag.

[10] Antoni ML, Scherptong RWC, Atary JZ, Boersma E, Holman ER, Wall EEVD (2010). Prognostic value of right ventricular function in patients after acute myocardial infarction treated with primary percutaneous coronary intervention. Circulation Cardiovascular Imag.

[11] Ancona F, Melillo F, Calvo F, Attalla El Halabieh N, Stella S, Capogrosso C (2021). Right ventricular systolic function in severe tricuspid regurgitation: prognostic relevance of longitudinal strain. Europ Heart J Cardiovas Imag.

[12] Fine NM, Chen L, Bastiansen PM, Frantz RP, Pellikka PA, Oh JK (2013). Outcome prediction by quantitative right ventricular function assessment in 575 subjects evaluated for pulmonary hypertension. Circulat Cardiovas Imag.

[13] Stolfo D, Albani S, Biondi F, De Luca A, Barbati G, Howard L (2020). Global right heart assessment with speckle-tracking imaging improves the risk prediction of a validated scoring system in pulmonary arterial hypertension. J Am Soc Echocardiogr.

[14] Park JH, Park JJ, Park JB, Cho GY (2018). Prognostic value of biventricular strain in risk stratifying in patients with acute heart failure. J Am Heart Assoc.

[15] Park J-H, Park MM, Farha S, Sharp J, Lundgrin E, Comhair S (2015). Impaired global right ventricular longitudinal strain predicts long-term adverse Outcomes in patients with pulmonary arterial hypertension. JCU.

[16] Skaarup KG, Lassen MCH, Johansen ND, Olsen FJ, Lind JN, Jørgensen PG (2021). Age- and sex-based normal values of layer-specific longitudinal and circumferential strain by speckle tracking echocardiography: the Copenhagen city heart study. Europ Heart J Cardiovas Imag.

[17] Skaarup KG, Lassen MCH, Marott JL, Biering-Sørensen SR, Jørgensen PG, Appleyard M (2020). The impact of cardiovascular risk factors on global longitudinal strain over a decade in the general population: the copenhagen city heart study. Int J Cardiovasc Imaging.

[18] Muraru D, Onciul S, Peluso D, Soriani N, Cucchini U, Aruta P (2016). Sex- and method-specific reference values for right ventricular strain by 2-dimensional speckle-tracking echocardiography. Circulation Cardiovascular Imaging..

[19] Park J-H, Choi J-O, Park SW, Cho G-Y, Oh JK, Lee J-H (2018). Normal references of right ventricular strain values by two-dimensional strain echocardiography according to the age and gender. Int J Cardiovasc Imaging.

[20] Fine NM, Chen L, Bastiansen PM, Frantz RP, Pellikka PA, Oh JK (2015). Reference values for right ventricular strain in patients without cardiopulmonary disease: a prospective evaluation and meta-analysis. Echocardiography.

[21] Addetia K, Miyoshi T, Citro R, Daimon M, Gutierrez Fajardo P, Kasliwal RR (2021). Two-dimensional echocardiographic right ventricular size and systolic function measurements stratified by sex, age, and ethnicity: results of the world alliance of societies of echocardiography study. J Am Soc Echocardiogr.

[22] Wang TKM, Grimm RA, Rodriguez LL, Collier P, Griffin BP, Popović ZB (2021). Defining the reference range for right ventricular systolic strain by echocardiography in healthy subjects: a meta-analysis. PLoS ONE.

[23] Chia E-M, Hsieh CHC, Boyd A, Pham P, Vidaic J, Leung D (2014). Effects of age and gender on right ventricular systolic and diastolic function using two-dimensional speckle-tracking strain. J Am Soc Echocardiogr.

[24] Carluccio E, Biagioli P, Alunni G, Murrone A, Zuchi C, Coiro S (2018). Prognostic value of right ventricular dysfunction in heart failure with reduced ejection fraction. Circul Cardiovas Imag.

[25] Kovács A, Lakatos B, Tokodi M, Merkely B (2019). Right ventricular mechanical pattern in health and disease: beyond longitudinal shortening. Heart Fail Rev.

