# Supplementary material for: Normal age- and sex-based values of right ventricular free wall and four-chamber longitudinal strain by speckle-tracking echocardiography: from the Copenhagen City heart study
Source: Clin Res Cardiol. 2023 Nov 15;113(3):456–68. doi: 10.1007/s00392-023-02333-x (PMC10881734; doi:10.1007/s00392-023-02333-x)
Supplement: Supplementary file 1 — Supplementary file1 (DOCX 27 kb) [file 392_2023_2333_MOESM1_ESM.docx]

**Supplemental Material**

”Normal age- and sex-based values of right ventricular free wall and four-chamber longitudinal strain by speckle-tracking echocardiography: From the Copenhagen City Heart Study”

Table of Contents

[1. Definitions of baseline comorbidities 2](#_Toc144901942)

[2. Additional information on right ventricular conventional parameters on echocardiography 2](#_Toc144901943)

[3. Reproducibility 3](#_Toc144901944)

[4. Table S1. Clinical and echocardiographic parameters associated with RV four-chamber longitudinal strain. 4](#_Toc144901945)

# **Definitions of baseline comorbidities**

Ischemic heart disease covered both a diagnosis of stable angina pectoris, chronic ischemic heart disease or a history of acute myocardial infarction.

Hypertension was defined as ICD-8/10 diagnostic code for hypertension, use of antihypertensive medication, systolic blood pressure ≥140 mmHg and/or diastolic blood pressure ≥90 mmHg based on the mean value of three blood pressure measurements at the study visit. Diabetes was defined as ICD-8/10 diagnostic code for diabetes or self-reported use of antidiabetic medication. Information on use of heart medication was obtained from the self-administered questionnaire.

# **Additional information on right ventricular conventional parameters on echocardiography**

TAPSE was measured with M-mode by placing the cursor over the lateral tricuspid valve annulus. FAC was calculated as the difference between the end-diastolic and end-systolic RV area relative to the end-diastolic RV area. RV S’ was measured with pulsed-wave tissue Doppler imaging by placing the cursor over the lateral annulus of the tricuspid valve. RVSP was calculated using the Bernoulli equation from a continuous-wave Doppler sample at the tricuspid regurgitant jet and an estimate of the right atrial pressure based on the inferior vena cava diameter and collapsibility.

# **Reproducibility**

In terms of intra-reader agreement, mean difference was 0.35 (95% limits of agreement: -3.31, 4.01) and -0.07 (95% limits of agreement: -2.84, 2.71) for RVFWLS and RV4CLS, respectively. The corresponding ICC for RVFWLS and RV4CLS were 0.94 and 0.91, respectively. In terms of inter-reader agreement, mean difference was -0.19 (95% limits of agreement: -5.45, 5.07) and -0.17 (95% limits of agreement: -4.42, 4.07) for RVFWLS and RV4CLS, respectively. The corresponding ICC for RVFWLS and RV4CLS were 0.85 and 0.83, respectively.

# **4. Table S1. Clinical and echocardiographic parameters associated with RV four-chamber longitudinal strain.**

|  | **Univariable** | | **Multivariable*** | |
| --- | --- | --- | --- | --- |
|  | **Standardized ß-coefficient (95% CI)** | **P** | **Standardized ß-coefficient (95% CI)** | **P** |
| **Clinical parameters** |  |  |  |  |
| Age, years | -0.05 | 0.11 | -0.04 | 0.27 |
| Systolic blood pressure, mmHg | -0.17 | <0.001 | -0.08 | 0.05 |
| BMI, kg/m^2^ | -0.21 | <0.001 | -0.13 | <0.001 |
| Total cholesterol, mmol/L | -0.08 | 0.004 | -0.04 | 0.31 |
| Heart rate, bpm | -0.04 | 0.12 | 0.01 | 0.72 |
| **Echocardiographic parameters** |  |  |  |  |
| LVEF, % | 0.22 | <0.001 | 0.18 | <0.001 |
| GLS, % | 0.40 | <0.001 | 0.33 | <0.001 |
| LVMI, g/m^2^ | -0.11 | <0.001 | 0.03 | 0.50 |
| LAVI, mL/m^2^ | 0.01 | 0.68 | 0.04 | 0.35 |
| E/A | 0.12 | <0.001 | 0.05 | 0.38 |
| E/e’ | -0.01 | 0.78 | -0.08 | 0.08 |
| TR velocity, m/s | -0.07 | 0.07 | -0.02 | 0.78 |
| TAPSE, mm | 0.22 | <0.001 | 0.26 | <0.001 |
| S’, m/s | 0.22 | <0.001 | 0.25 | <0.001 |
| FAC, % | 0.10 | 0.001 | 0.09 | 0.02 |
| RVSP, mmHg | 0.10 | 0.005 | 0.12 | 0.001 |

*BMI: body mass index, LVEF: left ventricular ejection fraction, GLS: global longitudinal strain, LVMI: left ventricular mass index, LAVI: left atrial volume index, DT: deceleration time, TR: tricuspid regurgitation, TAPSE: tricuspid annular plane systolic excursion, FAC: fractional area change, RVSP: right ventricular systolic pressure.*

*Adjusted for age, gender, smoking status, physical activity, heart rate, systolic blood pressure, RVSP and LVEF.

**Strain values were considered as absolute values.
